# Antimicrobial Susceptibility Profile of Several Bacteria Species Identified in the Peritoneal Exudate of Cows Affected by Parietal Fibrinous Peritonitis after Caesarean Section

**DOI:** 10.3390/vetsci8120295

**Published:** 2021-11-29

**Authors:** Salem Djebala, Julien Evrard, Fabien Gregoire, Calixte Bayrou, Linde Gille, Justine Eppe, Hélène Casalta, Vincent Frisée, Nassim Moula, Arnaud Sartelet, Damien Thiry, Philippe Bossaert

**Affiliations:** 1 Clinical Department of Production Animals, Faculty of Veterinary Medicine, University of Liège, Quartier Vallée 2, Avenue de Cureghem 7A-7D, 4000 Liège, Belgium; calixte.bayrou@uliege.be (C.B.); linde.gille@uliege.be (L.G.); Justine.eppe@uliege.be (J.E.); hcasalta@uliege.be (H.C.); vfrisee@uliege.be (V.F.); asartelet@uliege.be (A.S.); p.bossaert@uliege.be (P.B.); 2 Gestion et Prévention de Santé, Regional Association of Health and Animal Identification, Allée des Artisans 2, 5590 Ciney, Belgium; julien.evrard@arsia.be (J.E.); fabien.gregoire@arsia.be (F.G.); 3 Department of Veterinary Management of Animal Resources, Faculty of Veterinary Medicine, Fundamental and Applied Research for Animal & Health (FARAH), University of Liège, 4000 Liège, Belgium; nassim.moula@uliege.be; 4 GIGA—Animal Facilities—ULiège—B 34, 4000 Liège, Belgium; 5 Bacteriology, Department of Infectious and Parasitic Diseases, Faculty of Veterinary Medicine, University of Liège, Quartier Vallée 2, Avenue Cureghem 6, 4000 Liège, Belgium; damien.thiry@uliege.be

**Keywords:** parietal fibrinous peritonitis, caesarean section, peritoneal fluids, bacterial culture, antimicrobial susceptibility

## Abstract

The aim of this study was to identify the species and antimicrobial susceptibility of bacteria involved in parietal fibrinous peritonitis (PFP). We studied 156 peritoneal fluid samples from cows presenting PFP after caesarean section. Bacteria were cultured in selective media and their antimicrobial susceptibility was tested by disk diffusion assay. Bacteria were isolated in the majority (129/156; 83%) of samples. The majority (82/129; 63%) of positive samples contained one dominant species, while two or more species were cultured in 47/129 (36%) samples. *Trueperella pyogenes* (*T. Pyogenes)* (107 strains) was the most identified species, followed by *Escherichia coli* (*E. coli*) (38 strains), *Proteus mirabilis* (*P. mirabilis*) (6 strains), and *Clostridium perfringens* (*C. perfringens*) (6 strains). Several other species were sporadically identified. Antimicrobial susceptibility was tested in 59/185 strains, predominantly *E. coli* (38 strains) and *P. mirabilis* (6 strains). Antibiotic resistance, including resistance to molecules of critical importance, was commonly observed; strains were classified as weakly drug resistant (22/59; 37%), multidrug resistant (24/59; 41%), extensively drug resistant (12/59; 20%), or pan-drug resistant (1/59; 2%). In conclusion, extensive antibiotic resistance in the isolated germs might contribute to treatment failure. Ideally, antimicrobial therapy of PFP should be based upon bacterial culture and susceptibility testing.

## 1. Introduction

Parietal fibrinous peritonitis (PFP) is a common complication after laparotomy in cows. It is characterized by the accumulation of a considerable volume of inflammatory exudate and fibrin inside a thick capsule between the outer sheath of the parietal peritoneum and the abdominal muscular layers [1,2,3,4,5,6,7]. The voluminous mass protrudes into the abdominal cavity and compresses the digestive organs [4,5]. In Belgium, PFP is frequently encountered in rural veterinary practice due to the large number of caesarean sections (CS) in the Belgian blue breed [4,5,6,7,8]. PFP affects roughly 1% of cows after CS, occurs several weeks after surgery, and has a mortality rate of 13% [2,3,7]. Symptoms are variable and may include hyperthermia, anorexia, weight loss, visual abdominal distention, and colic [4,5,6,7,9,10].

PFP is generally considered a sterile inflammatory process [3,4,5,6,7,10], although research groups have recently challenged this assumption by isolating several aerobic and anaerobic bacteria from PFP fluids [4,5,6,7,11,12]. In the few scientific reports on the treatment of PFP, authors suggest a long period of antimicrobial therapy in combination with surgical drainage and daily flushing of the PFP cavity [3,4,5,6,11]. Antimicrobial treatment failure has been reported and may be due to the impermeability of PFP for antibiotics [9,11] or antimicrobial resistance [4].

Despite its importance for cattle veterinarians, the aetiology, pathogenesis, treatment, and prognosis of PFP are very scarcely documented, and the few reports published on the subject only include a small number of cattle [4,5,6,7,9,10,11]. In this study, we aimed to identify the different bacterial species and their antimicrobial susceptibility in a larger number of PFP cases in order to gain more insight into the aetiology and antimicrobial treatment options of this complication.

## 2. Materials and Methods

Between March 2017 and March 2019, the Clinical Department of Production Animals (Liège University) and the Regional Association of Animal Health and Identification (ARSIA) collaborated to motivate Belgian rural veterinarians to take peritoneal fluid samples from cows affected by PFP. All rural practitioners from the ARSIA database were contacted by e-mail, and instructions for diagnosis and sample collection were published on the ARSIA web site. The diagnosis was made by critical point in the anamnesis including recent CS, weight, and appetite loss and lack of treatment response. Clinical examination criteria were dehydration, fever, decrease of ruminal and intestinal motility, and restricted arm mobility during rectal palpation. Ultrasound examination was used to confirm the diagnosis. Indeed, the specific diagnostic criterion for PFP was the accumulation of an anechogenic fluid and echogenic fibrin strands within a hyperechogenic capsule between the parietal sheath of the peritoneum and the muscle layers. In some cases, PFP was confirmed by explorative laparotomy [4,5,6,7].

In each case, 10 mL of peritoneal fluid was aseptically collected via ultrasound-guided paracentesis before surgical drainage. Disinfection consisted of a protocol where the skin at the site of the paracentesis was shaved and scrubbed using povidone-iodine soap (7.5%) followed by alcohol (96°). Samples were then kept at 4 °C and dispatched to the lab for aerobic and anaerobic bacterial culture and disk diffusion assays.

The invasive procedures (paracentesis) were done in cases encountered in the field, primarily for diagnostic and therapeutic purposes. At no point did the research protocol interfere with treatment decisions and housing or management of the cows. Therefore, the animals in our study did not fall into the definition of an experimental animal, and no ethics approval was required.

The samples for aerobic culture were grown on Columbia agar, Gassner and Columbia/Nalidixic acid agar media (Thermo Fisher Scientific, Brussels, Belgium) at 37 ± 2 °C. Samples for anaerobic culture were grown under anaerobic conditions on Schaedler medium at 37 ± 2 °C. Two readings of each medium were performed at 18 to 24 h and 36 to 48 h of incubation. Bacterial identification was performed by the Maldi Biotyper (Bruker Daltonics, Bremen, Germany). The culture was considered negative if no bacterial growth was observed, and positive when one or several bacteria were found.

The antimicrobial susceptibility was tested for several isolated strains but not for anaerobic bacteria and *Trueperella pyogenes* (*T. pyogenes*) since susceptibility tests for these strains are technically complicated [13,14,15]. According to the laboratory’s protocols, a predefined set of molecules was tested per isolated strain, based on international standards [16]. In addition, according to national recommendations, antimicrobials of critical importance (latest generation quinolones and cephalosporins) were systematically tested in each strain.

Antimicrobial sensitivity was tested via the disk diffusion assay using the following protocol. The procedure is explained in detail elsewhere [17]: in brief, a fresh (<24 h) suspension of a pure bacterial culture produced by specific technology (Inoclic ND^®^, Montpellier, France) was inoculated at a concentration of 10^7^ CFU/mL in a Muller–Hinton agar, in which antimicrobial impregnated disks (I2A, Montpellier, France) were embedded. For colistin, a pre-diffusion method was used. In short, the bacterial inoculation was performed in Muller–Hinton agar plates in which impregnated disks of colistin were placed and allowed to diffuse during 2 h at 35°, as described by Yauri Condora and co-workers (2019) [18].

After 21 ± 3 h of incubation at 37 ± 2 °C, growth inhibition zones were automatically measured using the SIRscan 2000 ND^®^. Using a software package (SIRweb^TM^ ND^®^, Montpellier, France), results were compared to the reference values provided by the Antibiogram Committee of the French Society of Microbiology (2018) and the European Committee on Antimicrobial Susceptibility Testing (EUCAST) (2021) [19], and then bacteria were classified as sensitive (S), intermediate (I), and resistant (R) for each tested molecule.

Based on the combined results of individual antimicrobial susceptibility tests, bacteria received a global resistance score, i.e., weakly resistant (resistant to less than three antibiotic classes), multidrug resistant (resistant to three or more antibiotic classes), extensively drug resistant (resistant to all except one or two antibiotic classes) or pan-drug resistant (resistant all antibiotic classes) [20].

Statistical analyses were performed using SAS (2001). A Chi 2 test was used to achieve qualitative analysis in order to compare the results of bacterial culture (proportion of samples giving a positive, negative, and contaminated result; proportion of monoculture or polyculture results; proportion of aerobic and anaerobic species; and different species of isolated bacteria). A chi-square test was also used to compare the global antimicrobial resistance scores (weakly resistant, multidrug resistant, extensively drug resistant, and pan-drug resistant) per species. The cut-off of significance was fixed at *p* < 0.05.

## 3. Results

### 3.1. Sample Collection and Bacterial Culture

In total, 175 cases of PFP were submitted by 75 veterinarians on 133 Walloon farms. A number of samples were lost or discarded due to poor sample quality or conservation; finally, 156 peritoneal fluid samples were used for bacteriology.

Positive culture results were obtained in 129/156 samples, while 18/156 were negative (*p* < 0.001). If more than three colonies were detected (9/156 samples), the peritoneal sample was classified as contaminated [15]. Aerobic or facultative anaerobic bacteria were identified in all the positive cultures (129/129), while anaerobic bacteria were identified in only 13/129 samples and always associated with aerobic germs (*p* < 0.001).

As displayed in Table 1, 185 bacterial strains belonging to 21 species were identified. The most cultured species was *T. pyogenes* (107 strains) (*p* < 0.001), followed by *Escherichia coli* (*E. coli*) (38 strains) and *Proteus mirabilis* (*P. mirabilis*) (6 strains); *Clostridium perfringens* (*C. perfringens*) was the most frequently isolated anaerobic bacteria (6 strains).

In 63% of positive samples (82/129), one species was isolated; the most encountered species in monoculture was *T. pyogenes* (64/82 cases), followed by *E. coli* (5/82). In 36% (47/129) of PFP samples, multiple bacterial species were identified (*p* < 0.001). The majority of these samples (42/47) yielded two species, and the association of *T. pyogenes* and *E. coli* was most abundant (25/42).

### 3.2. Disk Diffusion Assay

Antimicrobial susceptibility was tested in 59/185 identified strains, belonging to 12/21 bacterial species, as displayed in Table 2. *E. coli* (38 times) and *P. mirabilis* (6 times) were the most tested bacteria (Table 2).

Global resistance scores using the classification by Magiorakos and co-workers (2012) [20] are displayed in Table 3. All bacteria strains that were regularly cultured showed variable degrees of antimicrobial resistance.

For *E. coli*, 38 strains were tested. Resistance was most frequently observed to amoxicillin (27/38) and tetracycline (26/38), followed by kanamycin (23/38), trimethoprim-sulfonamide (23/38), florfenicol (16/38) and ceftiofur (7/38). All isolated *E. coli* strains were completely sensitive for colistin (Table 2). Combined results show that 12/38 *E. coli* strains were weakly resistant, 18/38 multidrug resistant, and 8/38 extensively drug resistant (*p* < 0.001).

Six *P. mirabilis* strains were tested, all of which showed complete susceptibility for ceftiofur and cefquinome and complete resistance to tetracycline and colistin. Resistance rates for the other antimicrobials ranged from 1/6 for amoxicillin-clavulanate, florfenicol, and marbofloxacin, to 4/6 for trimethoprim-sulfonamide, kanamycin, and amoxicillin. *P. mirabilis* could be classified as multidrug resistant in 3/6 cases and extensively drug resistant in 2/6 cases.

All other strains showed varying degrees of resistance; two sporadically identified strains (*Actinobacillus rossii* and *Mannheimia varigena*) were sensitive to all tested antimicrobials, and one strain of *Providencia rettgeri* showed complete resistance for all tested molecules.

Altogether, in all 59 tested strains, 22 were classified as weakly resistant, 24 as multidrug resistant, 12 as extensively drug resistant, and one as pan-drug resistant (*p* < 0.001) (Table 3).

Among the antibiotics tested, observed resistance per molecule ranged from 0% for tulathromycin and gamitromycin to 69% for amoxicillin and tetracycline. It is important to note that resistances were also observed against antimicrobials of second or third generation cephalosporins, such as cefoxitin (1/4), cefquinome (10/59), ceftiofur (10/59), and against the latest generations of quinolones such as enrofloxacin (17/59) and marbofloxacin (13/59) (Table 2).

## 4. Discussion

Despite the numeric importance of PFP in the field, there is a lack of scientific knowledge about the aetiology, pathogenesis, and treatment of PFP [3,4,5,6,7,11]. This paper presents a dataset containing the largest number of PFP cases observed in the field thus far.

At least one bacterial species was identified in more than 80% of the tested samples in this study, adding further support that PFP is not a sterile process [4,5,6,11]. On the one hand, this number may be underestimated due to the limited sensitivity of bacteriological culture. In addition, it is very likely that several cows were treated with antimicrobials before the sampling, modifying the culture results and susceptibility patterns [4,5,11,21,22]. On the other hand, the number of septic peritoneal fluids could be overestimated following the possible risk of contamination during the sample taken by the different veterinarians, although a strict antisepsis had been advised and most cultures only yielded only one or two species, less indicative of a contaminated sample [5,17].

Aerobic bacteria were isolated far more frequently than anaerobic germs from PFP. This contrasts with a previous publication [23] where mainly anaerobic bacteria originating from the endogenous vaginal flora were isolated in the peritoneum during CS, indicating the incised uterus as the main source of bacterial contamination during CS and postoperative complications such as peritonitis. In our study, *T. pyogenes*, *E. coli*, *P. mirabilis*, and *C. perfringens* were the most frequently isolated species in PFP samples. These bacteria are ubiquitous in the environment [24] and can colonise a wide range of tissues and organs in cattle [21,25,26,27]. They are considered germs that can cause clinical infections in cases of severe contamination or immunosuppressive conditions such as calving and CS [26,28,29,30,31]. Altogether, our results suggest that bacterial contamination during CS can occur from endogenous sources, as stated elsewhere [23], but also from a wide variety of other sources, such as the environment, the surgeon’s hands, the surgical material or the cow’s skin. Interestingly, PFP occurs typically in association with specific farms and/or specific veterinarians [3,5,6,11], suggesting a high contamination risk (surgical technique, farm hygiene) or a low herd immunity status as major risk factors.

It should be noted that the low amount of isolated anaerobic bacteria in our study may be an underestimation due to culture difficulties. Indeed, the growth of anaerobic bacteria need an enriched medium and anaerobic conditions from the sampling to the culture process [32], which is difficult to control in this study.

It should be stressed that the presence of germs in PFP cows does not prove a causal mechanism. Their exact role in the pathogenesis of PFP requires further studies. The presence of peritoneal fluids in matched negative control cows could have shed more light on the importance of a positive bacteriology, but this was not feasible in the current study setup.

Antimicrobial resistance is a major issue in human and veterinary medicine [33]. Antimicrobial resistance can be natural; for instance, *P. rettgeri* is naturally resistant to ampicillin, first generation cephalosporins, colistin, gentamycin, and tobramycin [20,34], and *P. mirabilis* to colistin and tetracycline [34,35]. Antibiotic resistance can also be acquired, when a selective advantage of genetically mutant strains arises under pressure of an antibiotic [36,37]. Furthermore, resistance genes for different groups of antimicrobials can be transmitted within and between different bacterial species [38]. As a result, resistance patterns in commensal and pathogenic livestock germs are often a reflection of the antimicrobial classes commonly used in the sector [39].

The considerable degree of resistance observed in this study could be due to general widespread use of antimicrobials to treat common diseases [40], but may also be the short-term result of antimicrobials used during elective CS. Many of the molecules to which *E. coli* strains were resistant, such as amoxicillin and tetracycline, are also commonly used during CS [8]. Alternatively, resistance patterns observed in the bacteria in PFP may be the result of antimicrobials used to treat disease symptoms prior to PFP diagnosis and sampling [4,5,10,11]. It is known that ubiquitous bacterial species, such as those isolated in this study, easily acquire and spread resistance because of their omnipresence and high exposure to any antimicrobial treatment in a herd [41,42].

Several bacterial strains isolated from PFP were resistant to a wide range of molecules, including latest generation cephalosporins and quinolones, although their use in veterinary medicine has declined drastically over the last decade due to strict regulation by the Belgian expertise centre of Antimicrobial Consumption and Resistance in Animals (AMCRA) [39].

A limitation of our study is that sampling was done by different operators in the cows, who were likely treated by antibiotics before the final diagnosis of PFP. Moreover, antimicrobial susceptibility could not be tested for all cultured strains. Hence, the resistance pattern of *T. pyogenes*, the most commonly isolated bacteria, remains unknown. However, based on the few studies where an antibiogram was performed for *T. pyogenes*, we can assume that antimicrobial resistance is also present in this species [14,15,43]. Finally, the antibiotic susceptibility could have been performed using quantitative techniques rather than disk diffusion; this would be more accurate in determination of clinical efficacy of antibiotics against the identified bacterial strains. However, given the lack of clinical breakpoints even this technique would still lack the power to predict true in vivo resistance [44].

In reports on the treatment of PFP, antimicrobials are administered for long periods of time, besides surgical drainage and flushing of the PFP cavity [3,4,9,11]. Authors regularly report disappointing results of antimicrobial treatment and assume that this is caused by the impermeability of the PFP capsule to antimicrobials [9,11]. Our results indicate that therapy failure of PFP could also be attributed, at least partly, to antimicrobial resistance, since a majority of bacteria identified in PFP displayed moderate or extensive antimicrobial resistance.

## 5. Conclusions

In Belgian cattle veterinary practice, PFP is a frequent but poorly documented complication of abdominal surgery. In our study, the presence of bacteria could be demonstrated in the majority of peritoneal fluid samples of PFP cows. Aerobic or facultative anaerobic bacteria, the majority of which are opportunistic, were significantly more represented than anaerobic bacteria. Antimicrobial resistance, including resistance to molecules of critical importance, was common in the isolated bacterial strains. This study highlights the level of antimicrobial resistance in Walloon farms in general and in cows suffering from PFP in particular and provides new insights in the therapy of PFP. Ideally, antimicrobial treatment of PFP should be based on bacterial isolation and antimicrobial susceptibility testing.

## Figures and Tables

**Table 1:** The results of aerobic and anaerobic culture performed on the PFP fluid samples.

Aerobic/Facultative Anaerobic Bacteria Cultured	Number of Positive Samples	Anaerobic Bacteria Cultured	Number of Positive Samples
*Trueperella pyogenes*	107	*Clostridium perfringens*	6
*Escherichia coli*	38	*Fusobacterium necrophorum*	3
*Proteus mirabilis*	6	*Bacteroides* sp.	1
*Streptococcus uberis*	3	*Bacteroides fragilis*	1
*Helcococcus ovis*	2	*Helcococcus ovis*	1
*Mannheimia varigena*	2	*Peptoniphilus indolicus*	1
*Staphylococcus aureus*	2	/	/
*Streptococcus dysgalactiae*	2	/	/
*Providencia rettgeri*	2	/	/
*Proteus* sp.	2	/	/
*Proteus vulgaris*	1	/	/
*Helcococcus* sp.	1	/	/
*Salmonella typhimurium*	1	/	/
*Streptococcus mitis*	1	/	/
*Pseudomonas aeruginosa*	1	/	/
*Actinobacillus rossii*	1	/	/
contaminants	9	/	1
Total	172 strains	Total	13 strains

**Table 2:** The detailed antimicrobial susceptibility results observed in the bacteria identified in PFP.

Bacteria Tested	*E. coli* (38)			*P. mirabilis* (6)		*S. uberis* (3)		*P. rettgeri* (2)		*S. dysgalactiae* (2)		*M. varigena* (2)		*S. mitis* (1)		*S. aureus* (1)		*A. rossii* (1)		*P. sp* (1)			*P. aeruginosa* (1)		*S. typhimurium* (1)	
Antibiotics	S	R	I	S	R	S	R	S	R	S	R	S	R	S	R	S	R	S	R	S	R	I	S	R	S	R
amoxicillin-clavulanate	15	10	13	5	1	3	0	0	2	2	0	2	0	1	0	0	1	1	0	0	0	1	0	1	0	1
amoxicillin	11	27	0	2	4	/	/	0	2	/	/	2	0	/	/	/	/	/	/	0	1	0	0	1	0	1
cefquinome	30	8	0	6	0	3	0	1	1	2	0	2	0	1	0	0	1	1	0	1	0	0	1	0	1	0
ceftiofur	31	7	0	6	0	3	0	1	1	2	0	2	0	1	0	0	1	1	0	1	0	0	0	1	1	0
colistin	38	0	0	0	6	/	/	0	2	/	/	2	0	/	/	/	/	/	/	0	1	0	1	0	1	0
enrofloxacin	27	11	0	3	3	3	0	1	1	2	0	2	0	1	0	0	1	1	0	0	1	0	1	0	1	0
florfenicol	20	16	2	5	1	/	/	1	1	/	/	2	0	/	/	/	/	/	/	0	1	0	0	1	0	1
gentamicin	25	13	0	4	2	3	0	1	1	2	0	2	0	1	0	1	0	1	0	1	0	0	1	0	1	0
kanamycin	15	23	0	2	4	/	/	0	2	/	/	2	0	/	/	/	/	/	/	0	1	0	0	1	1	0
marbofloxacin	27	10	1	5	1	3	0	1	1	2	0	2	0	1	0	0	1	1	0	0	0	1	1	0	1	0
tetracycline	12	26	0	0	6	1	2	0	2	1	1	2	0	1	0	0	1	1	0	0	1	0	0	1	0	1
trimethoprim-sulfonamide	15	23	0	2	4	3	0	0	2	2	0	2	0	1	0	1	0	1	0	0	1	0	0	1	1	0
cephalexin	/	/	/	/	/	3	0	/	/	2	0	/	/	1	0	0	1	1	0	/	/	/	/	/	/	/
cefoxitin	/	/	/	/	/	3	0	/	/	/	/	/	/	/	/	0	1	1	0	/	/	/	/	/	/	/
erythromycin	/	/	/	/	/	1	2	/	/	2	0	/	/	0	1	1	0	1	0	/	/	/	/	/	/	/
lincomycin	/	/	/	/	/	1	2	/	/	2	0	/	/	1	0	1	0	1	0	/	/	/	/	/	/	/
oxacillin	/	/	/	/	/	3	0	/	/	2	0	/	/	0	1	0	1	1	0	/	/	/	/	/	/	/
penicillin	/	/	/	/	/	2	1	/	/	2	0	/	/	1	0	0	1	1	0	/	/	/	/	/	/	/
spiramycin	/	/	/	/	/	1	2	/	/	2	0	/	/	1	0	1	0	1	0	/	/	/	/	/	/	/
tildipirosin	/	/	/	/	/	/	/	/	/	/	/	/	/	/	/	/	/	1	0	/	/	/	/	/	/	/
tulathromycin	/	/	/	/	/	/	/	/	/	/	/	/	/	/	/	/	/	1	0	/	/	/	/	/	/	/
gamitromycin	/	/	/	/	/	/	/	/	/	/	/	/	/	/	/	/	/	1	0	/	/	/	/	/	/	/
Species susceptibility	266	174	16	32	40	36	9	6	18	27	1	24	0	12	2	5	10	15	0	3	7	2	5	7	8	4
Total of tested strains	456			72		45		24		28		24		14		15		15		12			12		12	

*E. coli*: *Escherichia coli*; *P. mirabilis*: *Proteus mirabilis*; *S. uberis*: *Streptococcus uberis*; *P. rettgeri*: *Providencia rettgeri*; *S. dysgalactiae*: *Streptococcus dysgalactiae*; *M. varigena*: *Mannheimia varigena*; *S. mitis*: *Streptococcus mitis*; *S. aureus*: *Staphylococcus aureus*; *A. rossii*: *Actinobacillus rossii*; *P. sp*: *Proteus sp*; *P. aeruginosa*: *Pseudomonas aeruginosa*; *S. typhimurium*: *Salmonella typhimurium*. S: sensitive; I: intermediate; R: resistant.

**Table 3:** Classification of the identified bacteria according to the degrees of resistance described by Magiorakos et al. (2012) [20].

Bacteria Tested (Number of Tests)	Weakly Resistant: Resistant to Less than Three Tested Antimicrobial Classes	Multidrug Resistant: Resistant to Three or More Tested Antimicrobial Classes	Extensively Drug Resistant: Resistant to All Except One or Two Antimicrobial Classes	Pandrug Resistant: Resistant to All Tested Antimicrobials	*p*-Value
*E. coli* (38)	12 (32%)	18 (47%)	8 (21%)	0	*p* < 0.001
*P. mirabilis* (6)	1 (17%)	3 (50%)	2 (33%)	0	/
*S. uberis* (3)	3 (100%)	0	0	0	/
*P. rettgeri* (2)	0	1 (50%)	0	1 (50%)	/
*S. dysgalactiae* (2)	2 (100%)	0	0	0	/
*M. varigena* (2)	2 (100%)	0	0	0	/
*S. mitis* (1)	1 (100%)	0	0	0	/
*S. aureus* (1)	0	1 (100%)	0	0	/
*A. rossii* (1)	1 (100%)	0	0	0	/
*P. sp* (1)	0	0	1 (100%)	0	/
*P. aeruginosa* (1)	0	0	1 (100%)	0	/
*S. typhimurium* (1)	0	1 (100%)	0	0	/
Total	22/59 (37%)	24/59 (41%)	12/59 (20%)	1/59 (2%)	*p* < 0.001

*E. coli*: *Escherichia coli*; *P. mirabilis*: *Proteus mirabilis*; *S. uberis*: *Streptococcus uberis*; *P. rettgeri*: *Providencia rettgeri*; *S. dysgalactiae*: *Streptococcus dysgalactiae*; *M. varigena*: *Mannheimia varigena*; *S. mitis*: *Streptococcus mitis*; *S. aureus*: *Staphylococcus aureus*; *A. rossii*: *Actinobacillus rossii*; *P. sp*: *Proteus sp*; *P. aeruginosa*: *Pseudomonas aeruginosa*; *S. typhimurium*: *Salmonella typhimurium*.

## Data Availability

Data is contained within the article.
